# Integration of biomedical engineering principles in nursing education curriculum: A perspective

**DOI:** 10.1016/j.jtumed.2024.06.005

**Published:** 2024-06-24

**Authors:** Sirwan K. Ahmed, Ribwar A. Mohammed

**Affiliations:** College of Nursing, University of Raparin, Rania, Sulaymaniyah, Kurdistan Region, 46012, Iraq

**Keywords:** Biomedical engineering, Nursing education, Nursing practice, Nursing simulation

Dear Editor,

Nursing education is currently facing a critical moment due to advancements in healthcare and technology.^1^^,^^2^ As the needs of the aging population and complex health issues continue to evolve, nurses must adapt and improve their skills to provide efficient and excellent healthcare. Biomedical engineering, which applies engineering principles to healthcare, offers a significant opportunity to enhance nursing education and practice.^3^^,^^4^ This letter explores the integration of biomedical engineering principles into nursing education, highlighting its potential to transform the nursing profession and improve patient outcomes.

Traditionally, nursing education has focused on developing clinical skills, critical thinking abilities, and providing high-quality patient care. However, the rapid pace of technological advancements in the healthcare industry demands that nurses possess a wider range of skills. Biomedical engineering principles encompass various fields, such as bioinformatics, biomedical instrumentation, and biomechanics. These disciplines can contribute to nursing education in several ways.

One way in which biomedical engineering principles can greatly enhance nursing education is through the development of sophisticated simulation technology. High-fidelity simulators allow nursing students to practice intricate procedures and scenarios in a controlled environment, strengthening their clinical competency and ability to make informed decisions. By incorporating biomedical engineering principles into the design of simulations, educators can enhance the authenticity and immersion of the learning experience, better preparing students for real-world clinical practice. Furthermore, understanding the concepts of biomedical engineering can provide valuable insights into the creation of cutting-edge healthcare devices that nurses encounter in their work. Nurses must comprehend the fundamental concepts and functionality of various technologies, including wearable devices for remote patient monitoring and robotic-assisted surgical systems, in order to effectively integrate these technologies into patient care. By incorporating biomedical engineering principles into the nursing curriculum, educators can ensure that students have the necessary knowledge and skills to navigate the ever-evolving field of healthcare technology.^5^ Moreover, integrating biomedical engineering principles into nursing education facilitates interdisciplinary collaboration and drives innovation.^6^ Nurses with a strong grasp of biomedical engineering principles collaborate effectively with engineers, physicians, and other healthcare professionals to develop and implement innovative technologies and treatment methods, advancing patient care and improving healthcare outcomes.

While incorporating biomedical engineering principles into nursing education offers numerous benefits, there are several challenges that must be addressed. One challenge is the need for faculty members with knowledge in both nursing and biomedical engineering. Nursing educators may require additional training and resources to effectively integrate these two disciplines and develop appropriate programs. Another challenge is the rapid pace of technological advancement, which requires continuous revisions of nursing courses to ensure relevance and currency. Nursing programs must establish mechanisms to evaluate and update their curricula consistently, incorporating advancements in technology and emerging developments in biomedical engineering.

Additionally, integrating biomedical engineering principles into nursing education may require modifications to accreditation standards and regulatory requirements. Nursing regulatory bodies and accrediting agencies should recognize the importance of incorporating technology and engineering principles into nursing programs. They should offer support and guidance to educational institutions seeking to adopt these modifications.

In conclusion, the incorporation of biomedical engineering principles into nursing education has significant potential for the future of the nursing profession. By equipping nurses with the necessary knowledge and skills to understand and utilize healthcare technologies, nursing education can be enhanced and students can be better prepared for the complexities and advantages of modern healthcare practice. However, successfully implementing this integration will require collaboration among educators, clinicians, engineers, and policymakers. This collaboration is necessary to overcome challenges and ensure that nursing programs remain responsive and adaptable to the evolving healthcare landscape. Through focused efforts and resource allocation, the integration of biomedical engineering concepts into nursing education can empower nurses to deliver exceptional care that is enhanced by technology. Ultimately, this will lead to improved patient outcomes.

## Source of funding

This research did not receive any specific grant from funding agencies in the public, commercial, or not-for-profit sectors.

## Conflict of interest

The authors have no conflict of interest to declare.

## Ethical approval

Ethical approval is not applicable for this article.

## Authors contributions

SKA and RAM conceived and wrote the entire article. All authors have critically reviewed and approved the final draft and are responsible for the content and similarity index of the manuscript.
